# Supplementary material for: scRegulate: Single-Cell Regulatory-Embedded Variational Inference of Transcription Factor Activity from Gene Expression
Source: bioRxiv. 2025 May 5:2025.04.17.649372. Preprint. [Version 2] doi: 10.1101/2025.04.17.649372 (PMC12248043; doi:10.1101/2025.04.17.649372)
Supplement: Supplement 1 [file media-1.pdf]

SUPPLEMENTARY MATERIAL

scRegulate: Single-Cell Regulatory-Embedded Variational Inference of Transcription Factor Activity from Gene Expression

Contents:

Note S1: Univariate Linear Model

Note S2: VAE Optimization

Note S3: Clustering Evaluation Metrics

Note S4: GRN Inference Performance Evaluation

Table S1: Dataset Details used in scRegulate

Fig. S1: Benchmarking of TFA embeddings using four datasets

Fig. S2: Benchmarking clustering performance robustness of TFA embeddings in dropout events

Fig. S3: Evaluation of scRegulate on MTG data

Fig. S4: Receiver Operating Characteristic (ROC) and Precision-Recall (PR) curves used for computing AUROC and AUPRC, respectively

Fig. S5: Benchmarking TFA inference using Perturb-seq data

Fig. S6: Clustering metrics across resolutions for RNA PCA, TF and latent embeddings

Fig. S7: UMAPs of the top 5 TFs per cell-type

Fig. S8: Cell-Type-Specific Regulatory and Coregulatory Networks

Disclaimer on the Use of Large Language Models

Supplementary Note S1: Univariate Linear Model

To initialize $\hat{\mathbf{e}}_{\mathrm{TF}}$, scRegulate employs a Univariate Linear Model (ULM) inspired by (Badia-i-Mompel *et al.*, 2022), where TF activities are estimated via:

$$\hat{\mathbf{e}}_{\mathrm{TF}}^{\mathrm{ULM}} = \mathbf{t}\left(\sum_{i=1}^{M} \mathbf{w}_{\mathrm{GRN},i} \mathrm{x}_i\right)$$

where $\mathbf{w}_{\mathrm{GRN},i}$ represents known regulatory interactions between all the TFs and gene i, $\mathbf{x}_i$ denotes gene expression value for the target gene i and $\mathbf{t}(.)$ computes the vector of t-statistics from the regression model. Briefly, this equation estimates TF activities as a weighted sum of all target gene expression values $\mathbf{x}_i$ in the prior GRN, where $\mathbf{w}_{\mathrm{GRN},i}$ represents the known regulatory weights for each of the M interactions.

Supplementary Note S2: VAE Optimization

The VAE is trained to minimize the evidence lower bound (ELBO):

$$\mathcal{L}_{ELBO} = \mathbb{E}_{q_{\boldsymbol{\phi}}(\mathbf{z}|\mathbf{x})}[\log p_{\boldsymbol{\theta}}(\mathbf{e}_{\mathrm{TF}}|\mathbf{z})] + \mathbb{E}_{p_{\boldsymbol{\theta}}(\mathbf{e}_{\mathrm{TF}}|\mathbf{z})}[log p_{\boldsymbol{\theta}}(\mathbf{x}|\mathbf{e}_{\mathrm{TF}})] - \beta D_{KL}(q_{\boldsymbol{\phi}}(\mathbf{z}|\mathbf{x}) \parallel p(\mathbf{z}))$$

$p_{\boldsymbol{\theta}}(\mathbf{e}_{\mathrm{TF}}|\mathbf{z})$ maps $\mathbf{z}$ to TF activities, $q_{\boldsymbol{\phi}}(\mathbf{z}|\mathbf{x})$ is the encoder distribution, $p_{\boldsymbol{\theta}}(\mathbf{x}|\mathbf{e}_{\mathrm{TF}})$ is the likelihood function, and β controls the strength of the Kullback-Leibler divergence between the encoder distribution and the prior $p(\mathbf{z})$ for latent space regularization.

The total loss is the sum of the Evidence Lower Bound (ELBO) and the GRN regularization loss:

$$\mathcal{L}_{\mathrm{Total}} = \mathcal{L}_{\mathrm{ELBO}} + \mathcal{L}_{\mathrm{GRN}}$$

Here $\mathcal{L}_{\mathrm{GRN}} = \gamma \sum_i \left\|\mathbf{w}_{\mathrm{GRN},i}\right\|_1$, where γ is the regularization parameter linearly scheduled over training epochs, and the sum is taken over all elements in $\mathbf{w}_{\mathrm{GRN},i}$, the vector of regulatory weights for gene i. The optimizer used for training is Adam, with an adaptive learning rate scheduler that dynamically adjusts based on validation loss. The initial learning rate is set to η_0 and is reduced according to:

$$\eta^{(n+1)} = \eta_0 \cdot \frac{1}{1 + \lambda t}$$

where λ controls the rate of decay, and t represents the iteration. Gradient clipping is applied with a maximum norm of 0.5 to stabilize training.

Unlike standard machine learning approaches that require explicit train-validation-test splits, scRegulate follows common scRNA-seq analysis practices, where benchmarking is performed using external reference datasets rather than separate test splits of the same dataset. To ensure model generalizability and prevent overfitting to noise, we apply an 85%-15% split between the training and validation subsets during model training. The validation loss is monitored during training, and an adaptive scheduler is used to adjust learning rates dynamically based on validation performance. Early stopping is triggered when no significant improvements are observed in validation loss over a set number of epochs, preventing unnecessary training cycles. No explicit test set is used from the same dataset, as benchmarking is performed using independent datasets for GRN inference and TF activity evaluation.

A global seed of 42 is used to ensure reproducibility across all computational steps.

Supplementary Note S3: Clustering Evaluation Metrics

The Adjusted Rand Index (ARI) measures clustering agreement while accounting for random chance:

$$ARI = \frac{\sum_{ij}\binom{n_{ij}}{2} - \left[\sum_i \binom{n_{i\cdot}}{2} \sum_j \binom{n_{\cdot j}}{2}\right] / \binom{N}{2}}{0.5\left[\sum_i \binom{n_{i\cdot}}{2} + \sum_j \binom{n_{\cdot j}}{2}\right] - \left[\sum_i \binom{n_{i\cdot}}{2} \sum_j \binom{n_{\cdot j}}{2}\right] / \binom{N}{2}}$$

where n_{ij} represents the number of samples assigned to both cluster i and true label j, $n_{i.}$ and $n_{.j}$ are the row and column sums of the contingency table, and N is the total number of samples.

The Normalized Mutual Information (NMI) evaluates clustering similarity based on information theory:

$$NMI = \frac{2I(U,V)}{H(U) + H(V)}$$

where $I(U,V)$ is the mutual information between predicted clusters U and true labels V, and $H(U)$, $H(V)$ are their respective entropies.

The F1-score quantifies the balance between precision and recall in cluster assignments:

$$F1 = \frac{2 \cdot Precision \cdot Recall}{Precision + Recall}$$

where precision and recall are defined as:

$$Precision = \frac{TP}{TP + FP}, Recall = \frac{TP}{TP + FN}$$

For multi-class clustering, the macro F1-score is computed as the average of the F1-scores over all classes:

$$Macro\ F1 = \frac{1}{K}\sum_{k=1}^{K} F1_k$$

where K is the number of cell types (clusters).

Supplementary Note S4: GRN Inference Performance Evaluation

GRN inference performance is evaluated using AUROC and AUPRC, computed via numerical integration:

$$AUROC = \int_0^1 TPR \quad dFPR \approx \sum_{i=1}^{1000} (FPR_i - FPR_{i-1})TPR_i$$

Here, TPR is the true positive rate, FPR is the false positive rate, and N (e.g., 1000) is the number of discrete steps used in the numerical approximation.

$$AUPR = \int_0^1 Precision \quad dTPR \approx \sum_{i=1}^{1000} (TPR_i - TPR_{i-1})Precision_i$$

This computes the area under the precision-recall curve, where the integration is approximated numerically over N steps.

To compare the similarity of inferred GRNs across cell types, Pearson correlation is computed between TF-target interaction matrices:

$$r_{W_{GRN}^{(c1)}, W_{GRN}^{(c2)}} = \frac{\sum (W_{GRN,ij}^{(c_1)} - \overline{W}_{GRN}^{(c_1)})(W_{GRN,ij}^{(c_2)} - \overline{W}_{GRN}^{(c_2)})}{\sum \left(W_{GRN,ij}^{(c_1)} - \overline{W}_{GRN}^{(c_1)}\right)^2 \sum \left(W_{GRN,ij}^{(c_2)} - \overline{W}_{GRN}^{(c_2)}\right)^2}$$

where $W_{GRN}^{(c1)}$ and $W_{GRN}^{(c2)}$ are the inferred GRN weight matrices for two different cell types, and $\overline{W}_{GRN}^{(c)}$ represents the mean regulatory weight per GRN.

Table S1: Dataset Details used in scRegulate

Dataset Name	Species	Source Name	Used in	Genes (post-QC)	Cells (post-QC)	Prior Used	# TFs retained	# Targets retained
Brain	Mus Musculus	Tabula Muris	Figure 2	18,631	3,401	collectri_mouse	264	4,768
Heart	Mus Musculus	Tabula Muris	Figure 2	13,646	624	collectri_mouse	222	3,748
Lung	Mus Musculus	Tabula Muris	Figure 2	15,960	5,449	collectri_mouse	245	4,281
PBMC	Homo Sapien	10X -scanpy	Figures 2, 4, 5	11,095	2,638	collectri_human	298	3,278
MTG	Homo Sapien	Allen Brain	Figure 2	36,601	137,303	collectri_human	459	5,981
PBMC	Homo Sapien	GRouNdGAN	Figure 3A	986	99,998	collectri_human	24	251
Tumor	Homo Sapien	GRouNdGAN	Figure 3A	836	100,000	collectri_human	36	276
Dahlin	Mus Musculus	GRouNdGAN	Figure 3A	971	100,000	collectri_mouse	22	323
Dixit*	Homo Sapien	Perturb-seq	Figure 3B	18,531	9,439	collectri_human	241	4,213

*This perturb-seq dataset, contains 10 TFs, each TF was targeted by multiple single-guide RNAs (gRNAs), and each gRNA was sequenced with both forward and reverse reads. To maximize robustness, we combined all gRNAs corresponding to the same transcription factor across their forward and reverse reads. To represent the perturbation of a given transcription factor, we identified cells that expressed at least two distinct gRNAs targeting the same TF (e.g., two different guides against CREB1). These were labeled as double knockdowns (e.g., CREB1 + CREB1) to enrich for stronger functional knockdown effects and reduce noise from single gRNA inefficiency. We included all available transcription factors with double knockdowns, namely ELK1, IRF1, EGR1, GABPA, E2F4, NR2C2, CREB1, ELF1, and ETS1. Cells with detected expression of only intergenic control gRNAs (non-targeting controls) were treated as baseline reference populations.

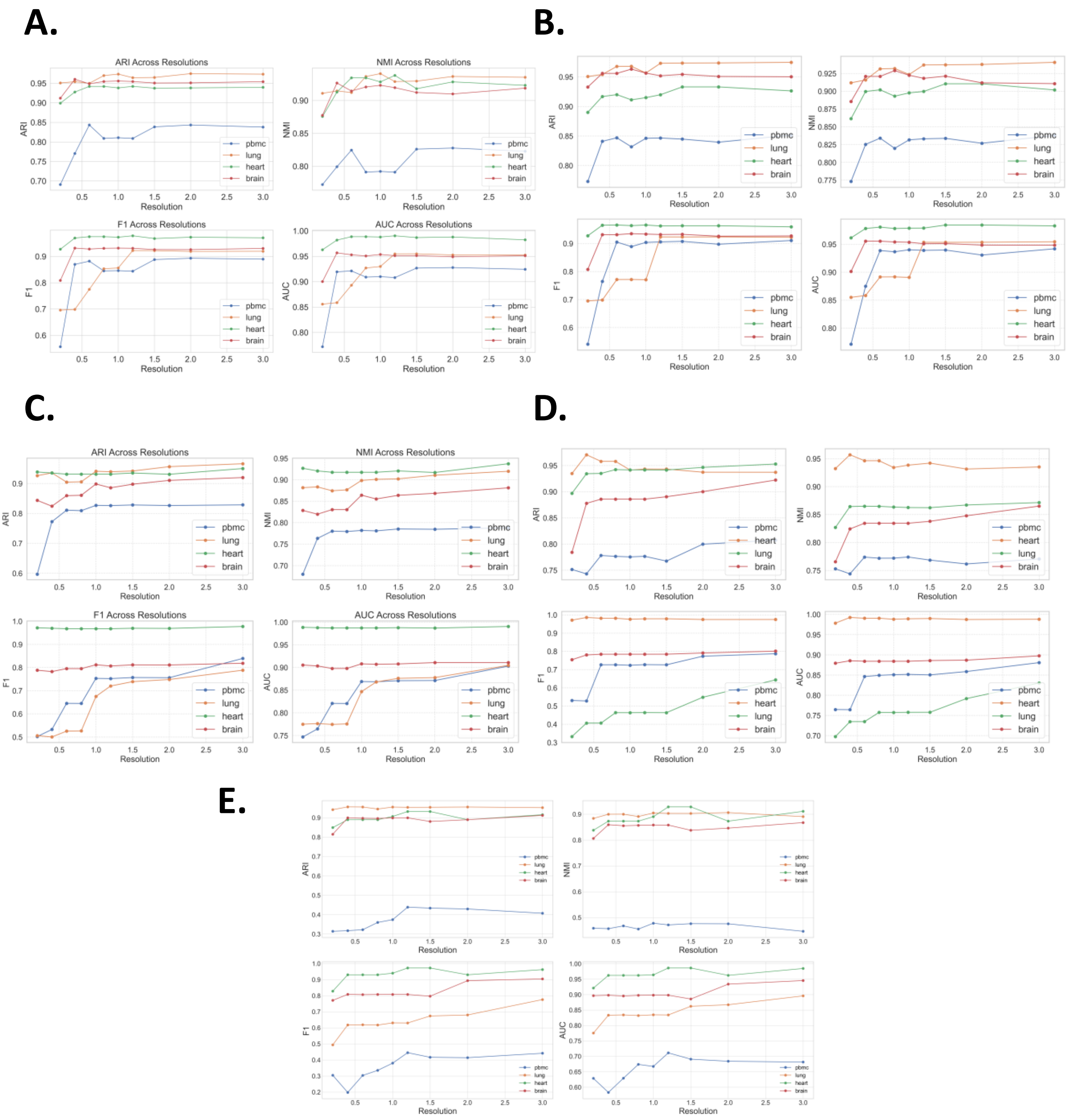

Fig S1. Benchmarking of TF activity embeddings using four datasets. For a wide range of the Leiden clustering resolutions, we computed different clustering metrics in (A) scRegulate, (B) decoupleR, (C) pySCENIC, (D) BITFAM and (E) BIOTIC.

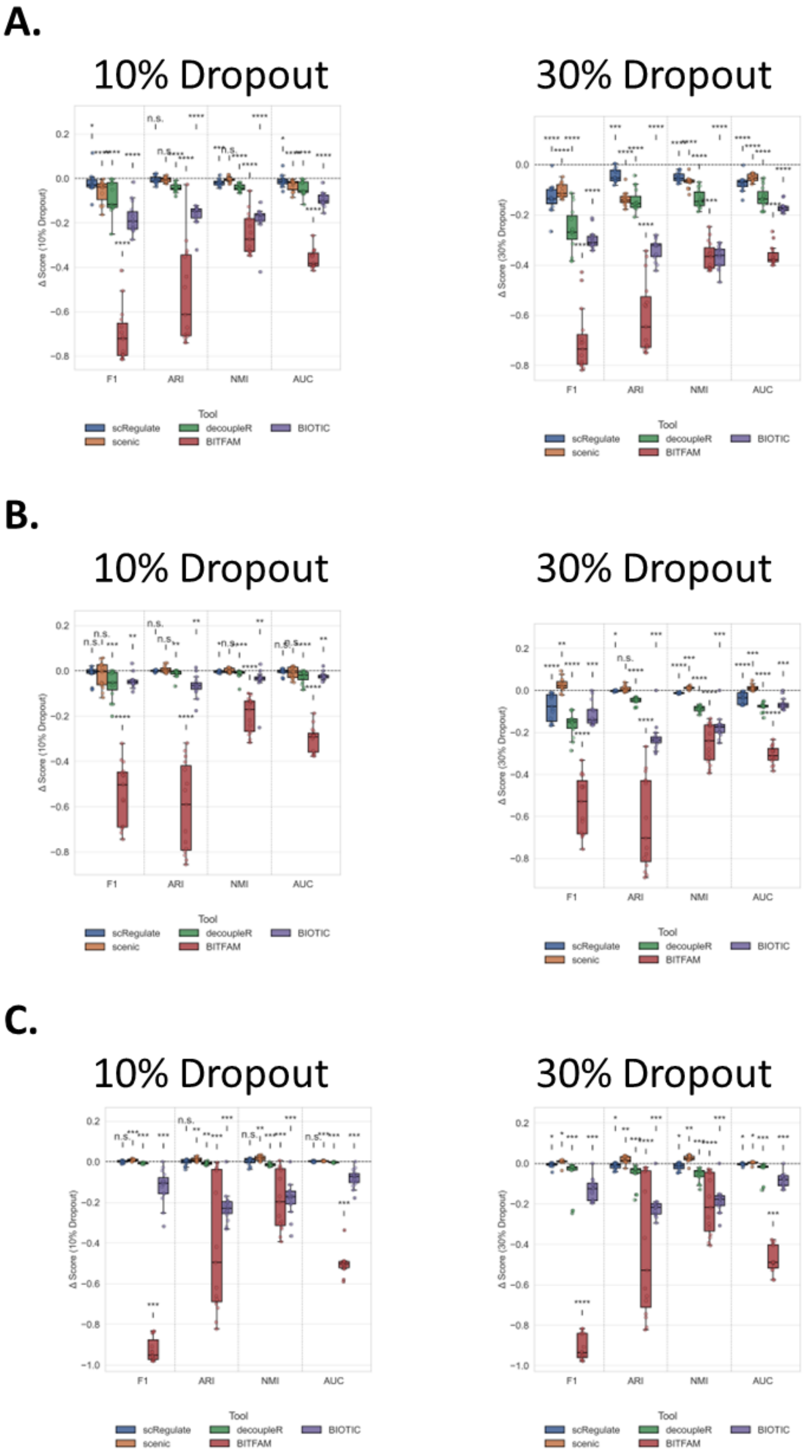

Fig S2. Benchmarking clustering performance robustness of TF Activity embeddings in dropout events. Each panel shows the drop in different clustering metrics colored by the tool applied for **(A)** PBMC **(B)** Lung and **(C)** Heart datasets. The drop between the original and noisy datasets in the evaluation metric is indicated by star(s) if statistically significant and otherwise labeled as n.s.

A.

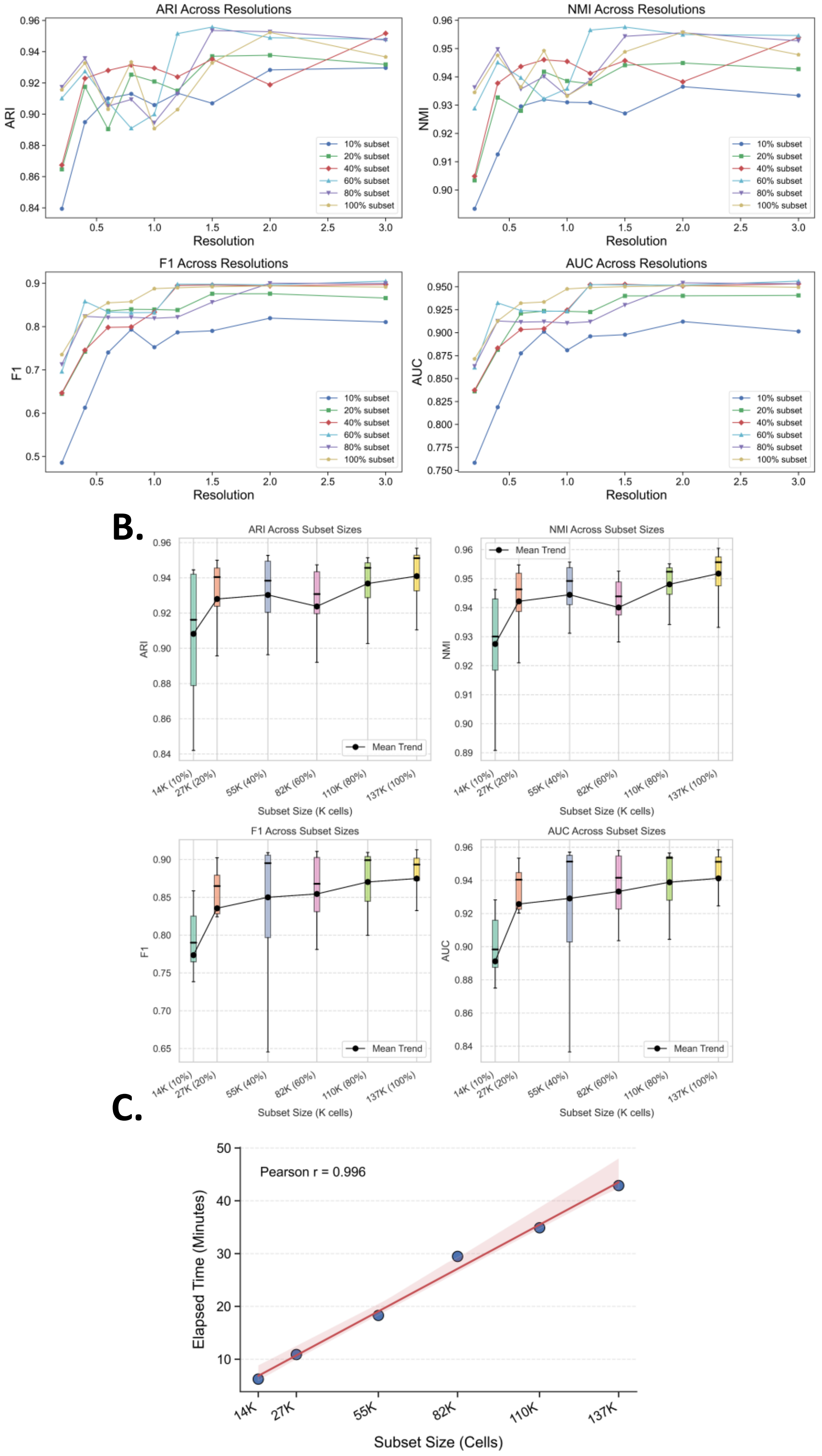

Fig S3. Evaluation of scRegulate on MTG data. (A) Shows the clustering evaluation metrics (ARI, NMI, F1 and AUC) for a wide range of the Leiden clusters (resolutions) **(B)** shows the trend of the metrics in (A), and **(C)** Training time vs. dataset size exhibit linear trend $O(n)$.

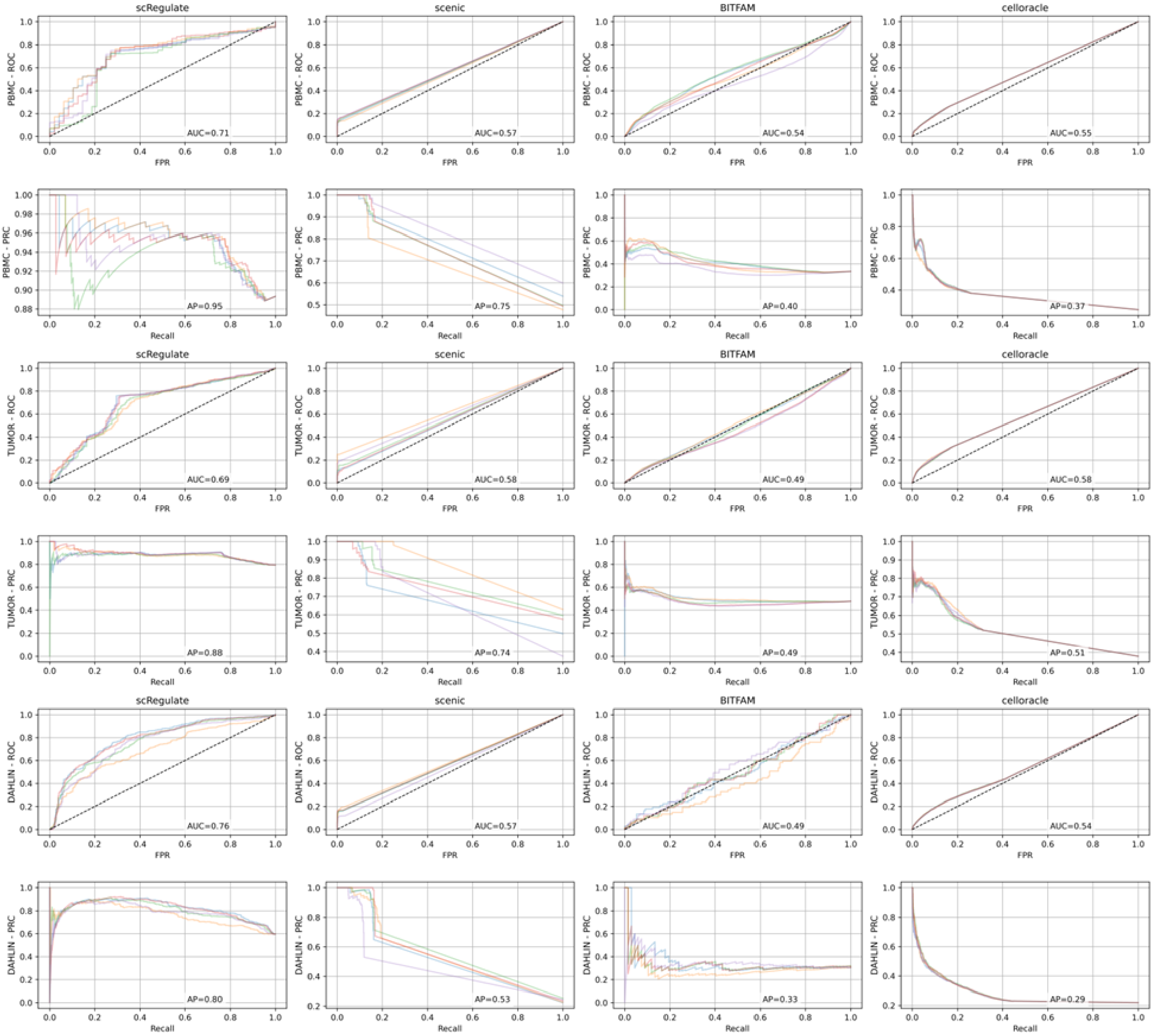

Fig S4. Receiver Operating Characteristic (ROC) and Precision-Recall (PR) curves used for computing AUROC and AUPRC, respectively. Columns correspond to the (A) scRegulate, **(B)** pySCENIC, **(C)** BITFAM, and **(D)** CellOracle tools. Rows Are ROC and PRC.

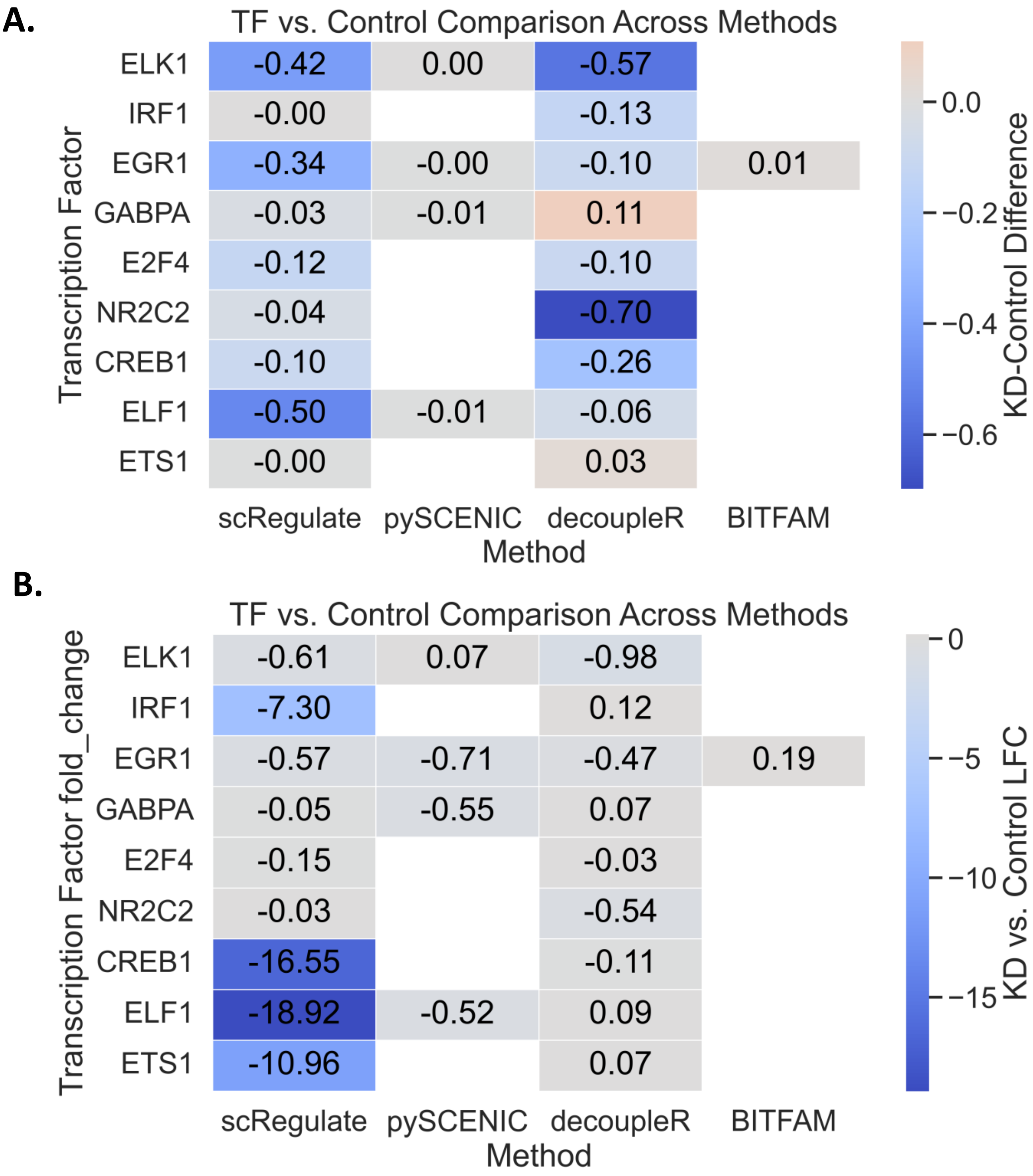

Fig S5. Benchmarking TF activity inference using Perturb-seq data. (A) Shows the absolute differences of inferred activities between knockdown vs. control samples. **(B)** shows the same comparison but based on log2fold change.

A.

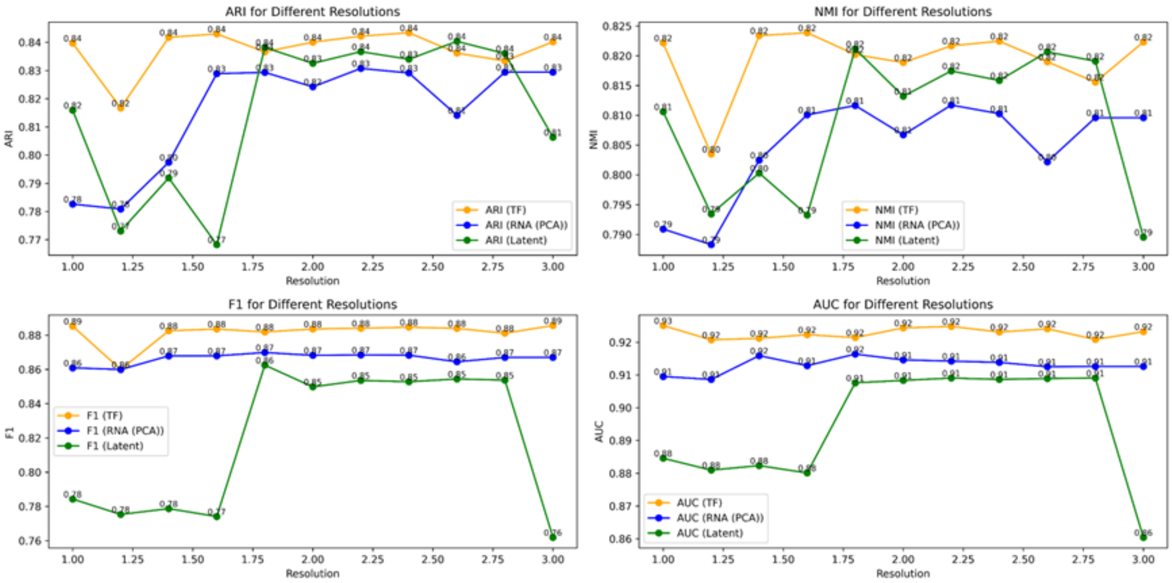

B.

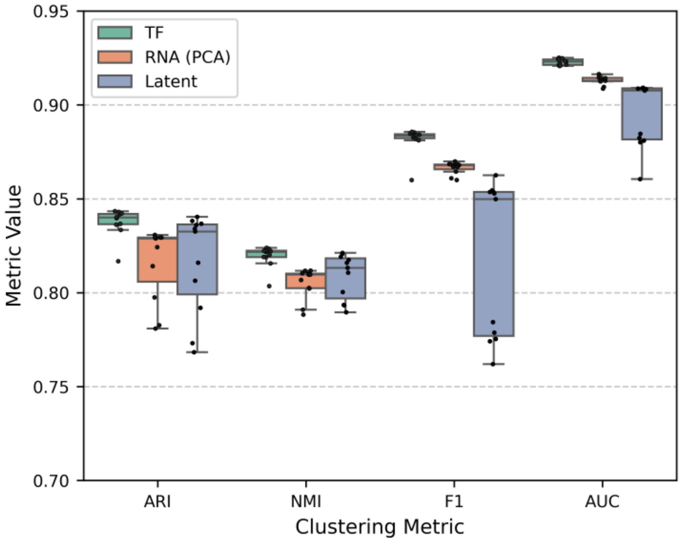

Fig S6. Clustering metrics across resolutions for RNA, TF and latent embeddings. (A) Post-training evaluation of the TF embedded layer with the latent representation and RNA modalities in PBMC Human dataset for various resolutions using Leiden clustering. TF embedding outperforms other modalities based on critical evaluation metrics of ARI, NMI, F1, and AUC. It also sows that the clustering stability and silhouette scores are competitive to the latent space. **(B)** scRegulate's nonlinear TF embedding achieves superior performance compared to PCA-based dimension reduction, as measured by ARI, NMI, F1 and AUC scores against ground truth labels.

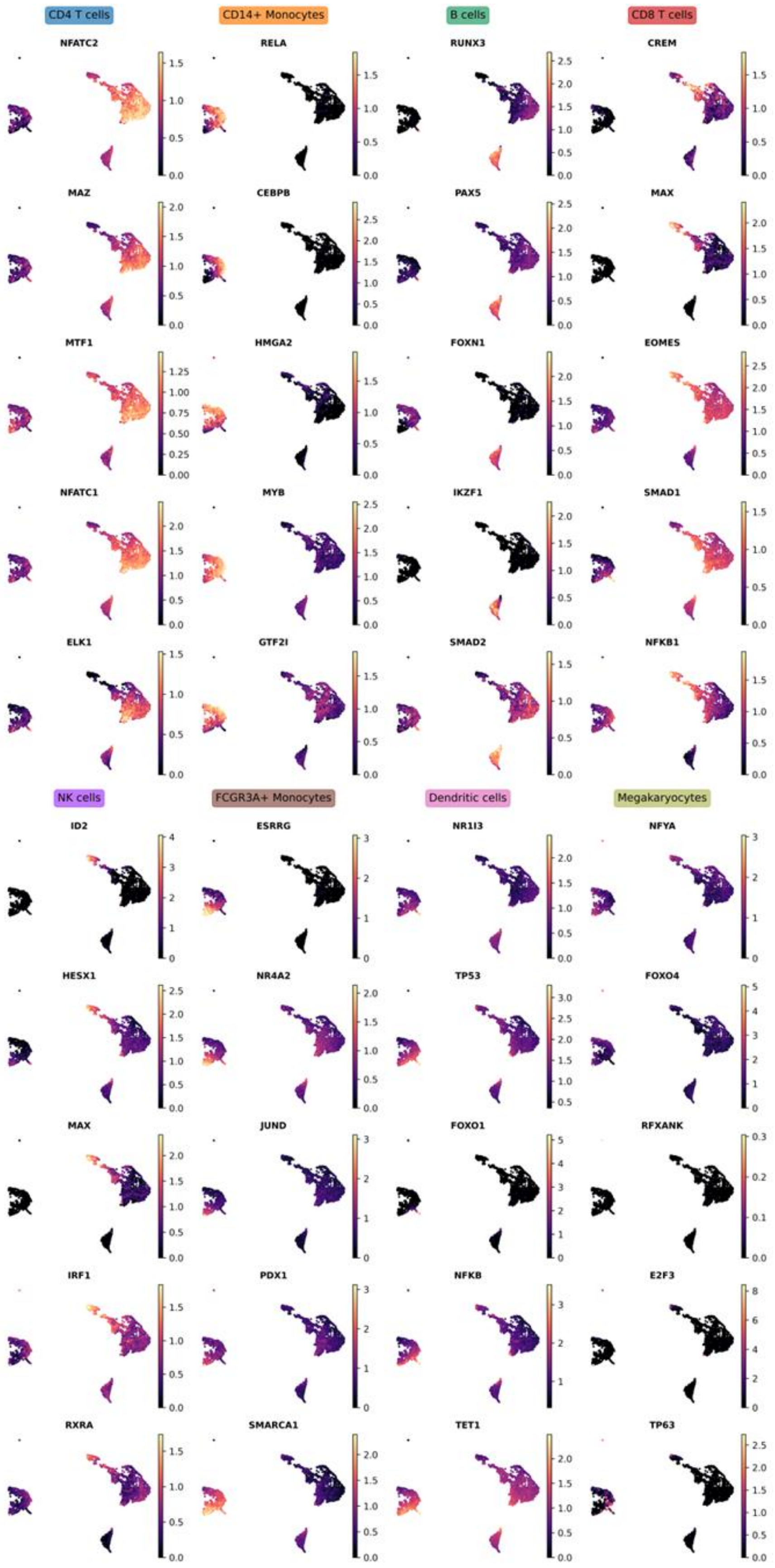

Fig S7. UMAPs of the top 5 TFs per cell-type shows scRegulate's capability of inferring cell-type specific TFs.

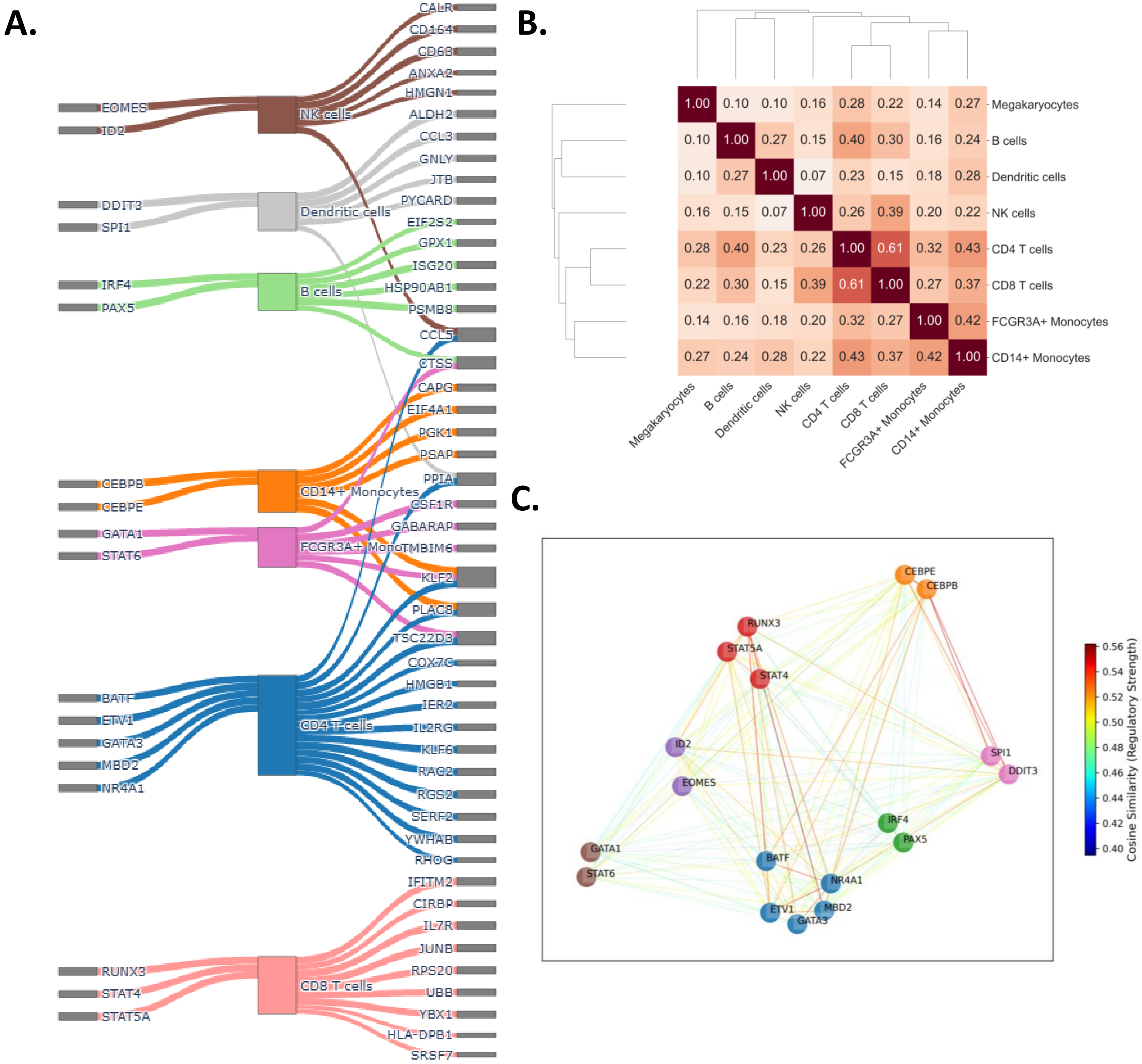

Fig. S8. Cell-Type-Specific Gene Regulatory and Co-regulatory Networks. (A) Sankey plot showing top differentially active transcription factors (TFs) (left) and their top three target genes based on the edge weights, highlighting key TF-target interactions. **(B)** Hierarchical clustering of cosine similarities (raised to the power of 8) between cell type-specific GRN matrices reveals biologically meaningful relationships, with T cells clustering closely together, while megakaryocytes stand out with markedly distinct regulatory patterns. **(C)** TF-TF coregulatory network of the TFs in panel C, with edge colors representing cosine similarity of target gene profiles and node colors representing cell-types. The GRN profiles were extracted from cell-specific networks.

Disclaimer on the Use of Large Language Models

ChatGPT (OpenAI) was used solely as a tool to refine and optimize code during the development of this study. It did not contribute intellectually to the study design, interpretation of results, or manuscript writing. All code outputs were manually reviewed, validated, and adapted by the authors. No text, figures, or other content in the manuscript was generated by ChatGPT. This usage complies with the acceptable use policy for large language models as defined by the International Society for Computational Biology (ISCB), and this statement is included in accordance with the journal's guidelines.
